# Robot-assisted distal gastrectomy with lymph node dissection for gastric cancer in a patient with situs inversus partialis: a case report with video file

**DOI:** 10.1186/s40792-018-0422-7

**Published:** 2018-02-13

**Authors:** Yuki Aisu, Yoshio Kadokawa, Shigeru Kato, Daiki Yasukawa, Yusuke Kimura, Tomohide Hori

**Affiliations:** Department of Gastrointestinal Surgery, Tenri Yorozu Sōdanjo Hospital, 200 Mishima-cho, Tenri City, Nara Prefecture 632-8552 Japan

**Keywords:** Cancer, Gastrectomy, Situs inversus, Robotic surgery, Robot

## Abstract

**Background:**

Situs inversus is a rare congenital condition that is currently classified into two types: complete situs inversus (situs inversus totalis, SIT) and partial situs inversus (situs inversus partialis, SIP). In SIP patients, some organs are inverted and others are in their expected position, and individual patient variation in organ position increases surgical difficulty. Several surgeons have performed laparoscopic or robotic surgeries in situs inversus patients, but almost all were SIT patients. We report the first case, to our knowledge, of an SIP patient with gastric cancer who was successfully treated by robot-assisted distal gastrectomy (RADG) with lymph node dissection.

**Case presentation:**

A 64-year-old woman diagnosed with early gastric cancer on the posterior midbody of the stomach was referred to our hospital for treatment. Computed tomography showed levocardia and inverted abdominal organs without enlarged lymph nodes or distant metastases. Polysplenia syndrome, intestinal malrotation, and left-sided gallbladder were also detected. RADG with D1+ lymph node dissection and Billroth I reconstruction (delta-shaped anastomosis) were performed using robotics. Hepatopathy caused by a liver retractor and pancreatic fistula were identified during the postoperative course, and the latter was classified as grade II based on Clavien-Dindo classification. The patient was discharged 18 days after the operation.

**Conclusions:**

Preoperative three-dimensional imaging is beneficial, and anatomical organ identification should be routinely performed, especially in SIP patients. We consider RADG a therapeutic option in SIP patients.

**Electronic supplementary material:**

The online version of this article (10.1186/s40792-018-0422-7) contains supplementary material, which is available to authorized users.

## Background

Situs inversus (SI) is a rare autosomal recessive congenital anomaly in which the major visceral organs are reversed or mirrored from their usual positions. The reported incidence rate is 0.005–0.02% [1, 2]. SI was first described in 1600 [1] and is currently classified into two types: complete situs inversus (situs inversus totalis, SIT) and partial situs inversus (situs inversus partialis, SIP). The thoracic and abdominal organs are completely reversed in SIT, whereas the organs are partially mirrored in SIP [3]. SIT occurs in approximately 90% of all SI cases; therefore, SIP is extremely rare [4].

Several surgeons have performed laparoscopic gastrectomy (LG) in SI patients [5–7], but all patients were categorized as SIT. Only one case of robot-assisted distal gastrectomy (RADG) in an SI patient has been reported, and robotic surgery is considered suitable only in SIT patients [8]. Any surgery in SIP patients is more complicated and technically difficult because of the unfamiliar anatomy [9–11]; therefore, almost all documented cases treated surgically have been SIT patients.

In SIP, some organs are inverted and others are in their expected position, and patient differences in organ position increase surgical difficulty [9–12]. We report the first case, to our knowledge, of RADG with intentional lymph node dissection in an SIP patient with gastric cancer. We also discuss the key points and pitfalls we encountered during RADG.

## Case presentation

**Fig. 1:**
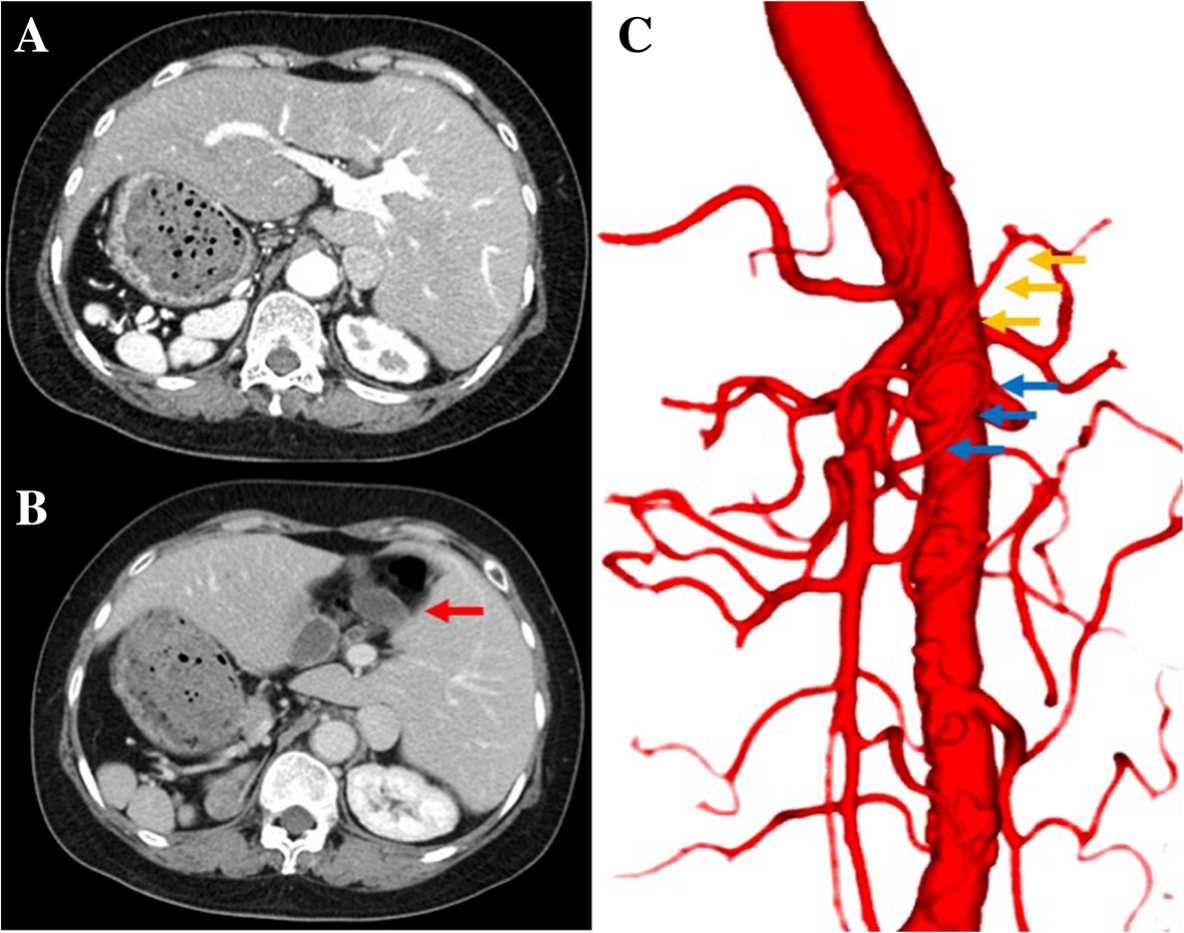
Computed tomographic (CT) image, preoperative three-dimensional CT angiography. **a**, **b** CT images showing polysplenia and left-sided gallbladder (*red arrow*). **c** Three-dimensional CT angiography showing the common hepatic artery (*yellow arrows*) and right gastroepiploic artery (*blue arrows*) arising from the first jejunal artery

A 64-year-old woman diagnosed with early gastric cancer was referred to our hospital for treatment. Gastrointestinal endoscopy showed an elevated lesion with ulcer (types 0–IIc) on the posterior midbody of the stomach, and histopathology revealed poorly differentiated adenocarcinoma. Computed tomography showed levocardia and inverted abdominal organs without enlarged lymph nodes or distant metastases. Polysplenia syndrome, intestinal malrotation, and left-sided gallbladder were detected. Well-known comorbidities associated with Kartagener syndrome, e.g., chronic sinusitis and bronchiectasis, were not seen, and a definitive diagnosis of SIP was made. The common hepatic and right gastroepiploic arteries both derived from the first jejunal artery (Fig. 1). Tumor marker levels were within normal ranges, her preoperative body mass index was 24.6 kg/m^2^, and preoperative diagnosis of gastric cancer was poorly differentiated adenocarcinoma of posterior midbody (0–IIc, cT1b N0 M0), categorized as stage IA according to both Japanese and tumor-node-metastasis (TNM) classifications [13, 14].

**Fig. 2:**
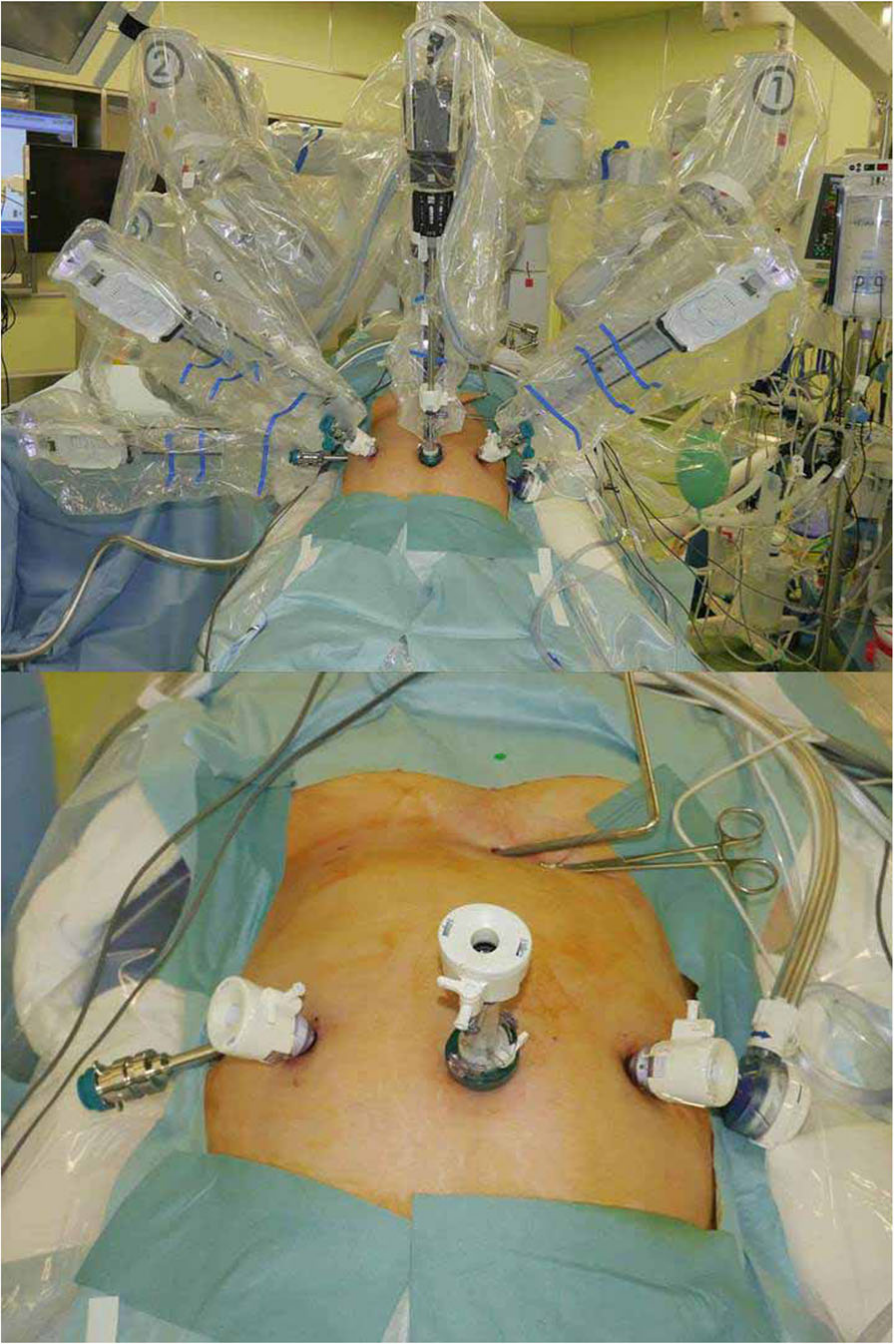
Placement of trocars and robotic arms. Photographs showing the surgical setup in our case

**Fig. 3:**
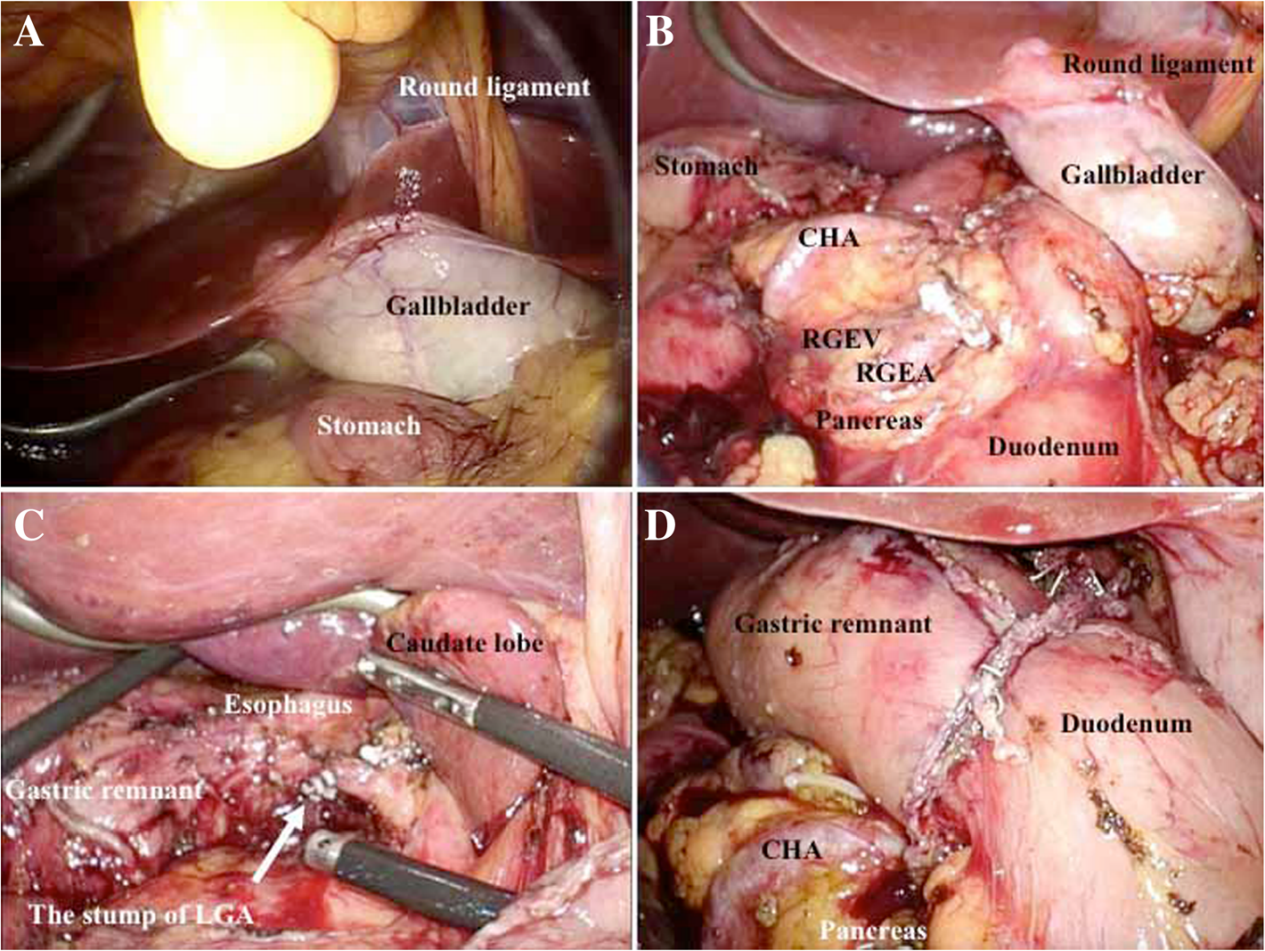
Intraoperative findings. **a** Laparoscopic view showing inversion of the abdominal organs. **b** After exposing the RGEA and RGEV on the left side, we completed the dissection of lymph node no. 6. **c** The LGA, which was located on the right side, was transected, and lymph node no. 7 and no. 9 were dissected. **d** Billroth I reconstruction was then performed. CHA common hepatic artery, RGEA right gastroepiploic artery, RGEV right gastroepiploic vein, LGA left gastric artery

According to Japanese guideline for gastric cancer treatment [15], optimal lymph node dissection was proposed as D1+ in this case. RADG with D1+ lymph node dissection and Billroth I reconstruction (delta-shaped anastomosis [16]) were performed using robotics (da Vinci Si Surgical System; Intuitive Surgical, Inc., Sunnyvale, CA, USA). Details of the RADG procedure are shown in Additional file 1: Video S1. Briefly, a three-dimensional camera was inserted into the abdominal cavity below the umbilicus. Four additional trocars were placed, then each robotic arm was docked (Fig. 2). The third robotic arm was usually docked to the patient’s left lateral trocar; however, SIP forced us to dock the third robotic arm to the right lateral trocar to address difficulties resulting from mirroring of the organs (Figs. 2 and 3). The patient-side surgeon improved the surgical field using the left lateral trocar, as needed. The round ligament of the liver was retracted ventrally using a suture carrier device (Endo Close; Medtronic, Minneapolis, MN, USA), and the left lobe was elevated with a Nathanson liver retractor (Cook Japan, Tokyo, Japan). D1+ lymph node dissection was performed based on the Japanese classification [13], and a total of 33 lymph nodes were examined. Surgical duration was 451 min, and blood loss was 150 ml.Additional file 1:**Video S1.** Actual procedures were shown in detail. (M4V 185053 kb)

Liver function tests indicated hepatopathy on postoperative day 1, and peak serum levels of aspartate aminotransferase, alanine aminotransferase, lactase dehydrogenase, total bilirubin, and γ-glutamyl transpeptidase were 1112 IU/L, 798 IU/L, 1406 IU/L, 15.4 μmol/L, and 14 IU/L, respectively. Hepatopathy resolved on postoperative day 5. A pancreatic fistula was also identified during the postoperative course and was classified as grade II based on Clavien-Dindo classification [17]. The patients’ postoperative hospital stay was 18 days. Histopathology identified moderately differentiated adenocarcinoma (0–IIc, 15 × 15 mm, pT1b (SM), int, INF-β, ly0, v0, pPM0, pDM0, pN0), categorized as stage IA, according to both Japanese and TNM classifications [13, 14]. As of this report, the patient is in good health with no recurrences.

## Discussion

SI has no effect on normal health or life expectancy [18] but is usually associated with certain anomalies, including ciliary dyskinesia and cardiac defects [12, 19, 20]; left-sided gallbladder, polysplenia, intestinal malrotation, and levocardia were confirmed in our case.

LG for gastric cancer was first performed in 1991 [21], and LG with lymph node dissection for advanced gastric cancer is now well developed in Japan [22] and considered safe and feasible [23, 24]. The first case of laparoscopic-assisted distal gastrectomy for SI patients with gastric cancer was reported in 2003 [25], and several cases have since been documented [5, 7, 25]. However, almost all were cases of SIT. SI increases surgical difficulty [26], and intraoperative anatomical recognition is especially difficult in SIP [12].

RADG for gastric cancer was first reported in Japan [27], and robotic gastrectomy for gastric cancer is currently used mainly in developed countries [28–30]. Compared with LG for gastric cancer, robotic gastrectomy has similar therapeutic potential for curative resectability and reduced postoperative stay [31, 32]. Regarding gastrectomy for gastric cancer, a robotic approach clearly has advantages including less blood loss and lower postoperative morbidity compared with LG, although robotic gastrectomy requires longer operative time and has lower cost-effectiveness [33, 34].

The first case of RADG for gastric cancer in SIT was reported in 2011 [8]. Our report is the first, to our knowledge, to describe robotic gastrectomy for gastric cancer in an SIP patient. As discussed, SIP increases surgical difficulty [9–12], and previous reports focused primarily on laparoscopic and robotic gastrectomies in SIT patients. In SIP patients, some organs are inverted and others are in their expected positions. Our SIP patient also had partial inversion, i.e., polysplenia, left-sided gallbladder, and bowel malrotation, increasing the surgical difficulty.

In previously documented cases of LG in SI patients, the main surgeon and first assistant were positioned on the side opposite their routine position, and the scopist stood between the patient’s legs. This position change reduces the advantages of the dominant arm, is unfamiliar, and increases surgeons’ stress. However, some suggest that changes in positions are not essential in robotic surgery [8]. Hence, in comparison with LG in SI patients, we consider that a familiarity of surgeons’ positions and a utility of dominant arm may be advantageous points during robotic surgery in SI patients. In our case, we successfully completed RADG in our usual positions. We suggest that robotic gastrectomy may be suitable for SIP patients to resolve the technical difficulties.

**Fig. 4:**
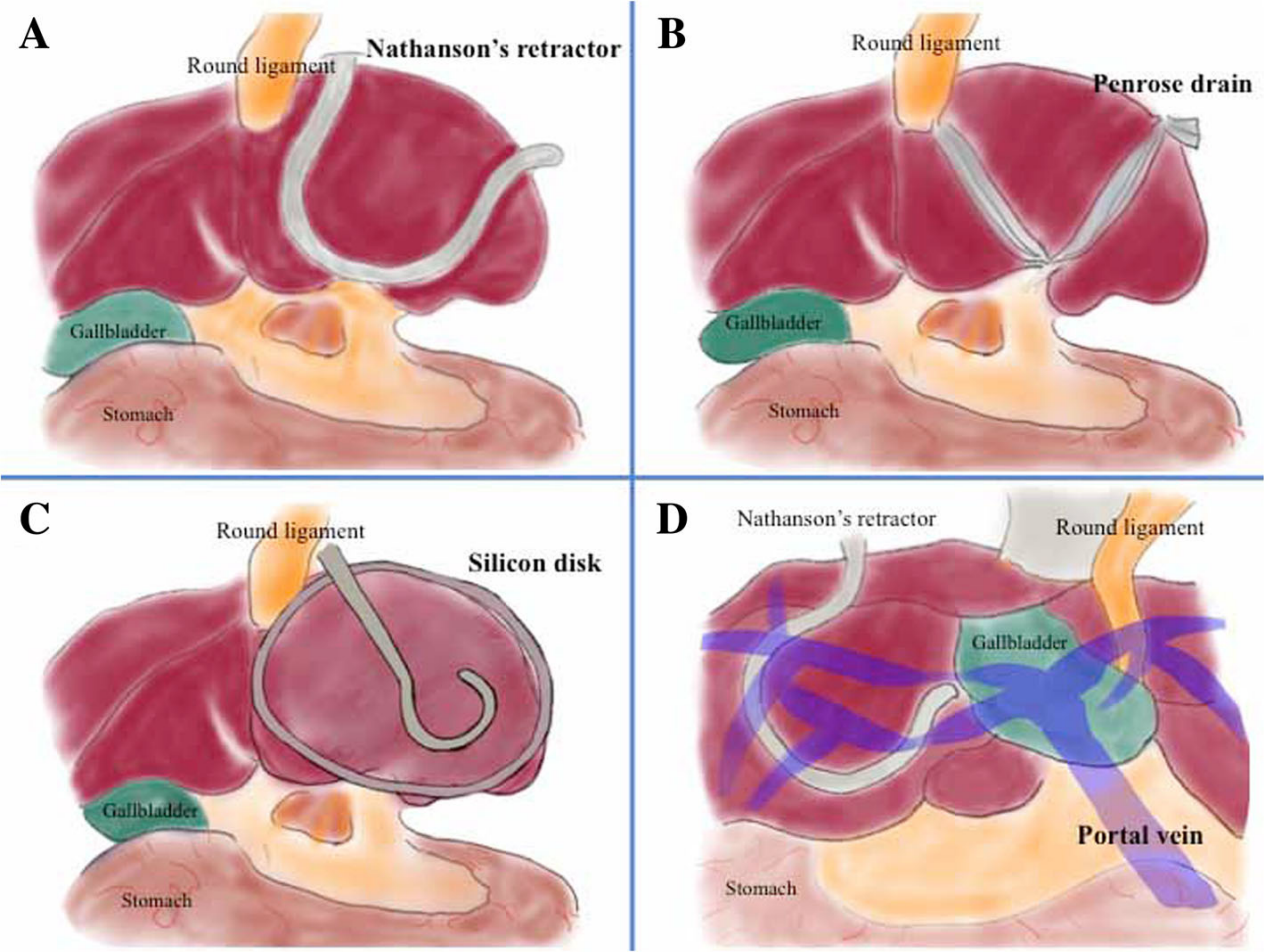
Methods of retracting the left liver lobe. The Nathanson liver retractor method (**a**), Penrose method (**b**), and silicon disk method (**c**) are shown. In our case (**d**), the first bifurcation of the portal vein was behind the gallbladder; therefore, the Nathanson retractor compressed the posterior and inferior branches of the portal vein

A Nathanson retractor, silicone disk, [35], or Penrose method [36] is used to fix the left liver lobe during LG to improve the surgical field (Fig. 4a–c). Robotic arm retraction is excessively forceful because robotic surgery involves no sense of touch. The Nathanson retractor has a large advantage that surgical field with an optimal liver retraction is excellent, because this retractor has no relation with robotic arm. We were concerned that compression by the robotic arm may cause unexpected liver damage; therefore, we used the Nathanson retractor. Although the Nathanson retractor is simple and easy to use, its use in our patient was technically difficult because of the left-sided gallbladder and anomalous arrangement of the portal vein (Fig. 4d); the anterior and posterior segment branches arose separately from the portal venous trunk on the extreme left side. Linear, not planar, compression may cause unexpected liver injury. Linear and edge compression to the central portal branches from the Nathanson retractor caused temporary liver ischemia intraoperatively in our case (Fig. 4d), which caused the remarkable elevations in serum liver enzymes in the early postoperative period. Planar compression using a silicone disk or Penrose drain may be the optimal method to prevent liver ischemia, although the Penrose method may be technically difficult in SI patients.

Simple question arose. Is there another possible explanation for postoperative liver dysfunction, such as gut ischemic change by pneumoperitoneum pressure or ligation of some hepatic branches because of abnormal anatomy? In our case, as shown in Fig. 4d, one specific feature of our SIP was characterized in the non-mirrored liver. This feature was summarized as SIP, not SIT. This non-mirrored liver caused technical difficulties and resulted in postoperative liver dysfunction, because the right-lobe liver itself should be retracted for surgical procedures related with mirrored organs (e.g., the esophagus, stomach, pancreas, and spleen). This liver retraction unfortunately caused a temporal occlusion of major portal branches.

Postoperative pancreatic fistula is an important complication in the field of gastrectomy. Recent reports have documented that robotic surgery can decrease pancreatic fistula in gastric surgery [37, 38]. However, pancreatic fistula was observed in our patient, and it was fortunately categorized as grade II in Clavien-Dindo classification. A possible explanation was that pancreatic injury during an intensive dissection of lymph nodes and/or a subtle retraction of the pancreas may occur. Especially, dissection of lymph node no. 6 was tough in our case, because the right gastroepiploic artery and the right gastroepiploic vein were located on the left side and accompanied with anomaly (Fig. 3b).

In our case, liver dysfunction and pancreatic fistula were observed after surgery, and these complications may be triggered by specific features of our SIP. We consider RADG a therapeutic option, even in SIP patients; however, anatomical identification based on detailed images should be routinely performed before robotic surgery in SIP patients. Three-dimensional images are useful to understand the vascular anatomy in SI, and vascular anomalies of the common hepatic and right gastroepiploic arteries were clearly detected in our case. Three-dimensional images are beneficial to identify vessel anomalies, and preoperative anatomical identification should be routinely performed, especially in SIP patients.

As described above, robotic surgery in SI patients may have some advantages (e.g., a familiarity of surgeons’ positions and a utility of dominant arm) in SI patients. Though we believe robotic surgeries potentially have substantial benefits, our speculation that robotic gastrectomy may be suitable for SIP patients to resolve the technical difficulties sounds exaggerated. In fact, we experienced only this SIP case who received robotic gastrectomy. Each country has own health insurance system, and Japanese government employs a universal health insurance system [39]. Though novel surgical procedures (e.g., robotic surgeries) were still not authorized in Japan, robotic gastrectomy will be listed in the health insurance system’s listing by the governmental council in April 2018. Paradoxically, if once listed in the health insurance system, Japanese surgeons have to serve patients with advanced techniques, though specific regulations and ethical policies should be respected [39]. We speculate RADG a therapeutic option, even in SIP patients, and hope our experience will be informative for surgeons around the world.

## Conclusions

Despite SIP, polysplenia, left-sided gallbladder, and bowel malrotation, we performed RADG successfully, in our patient. We report the first case, to our knowledge, of RADG with lymph node dissection in an SIP patient with gastric cancer.

## Abbreviations

LG: Laparoscopic gastrectomy
RADG: Robot-assisted distal gastrectomy
SI: Situs inversus
SIP: Situs inversus partialis
SIT: Situs inversus totalis
